# 2-methylbutyryl-CoA dehydrogenase deficiency associated with autism and mental retardation: a case report

**DOI:** 10.1186/1752-1947-1-98

**Published:** 2007-09-20

**Authors:** Oivind J Kanavin, Berit Woldseth, Egil Jellum, Bjorn Tvedt, Brage S Andresen, Petter Stromme

**Affiliations:** 1 Department of Pediatrics, Ullevål University Hospital, Oslo, Norway; 2 Department of Clinical Chemistry, Rikshospitalet-Radiumhospitalet Medical Center, Oslo, Norway; 3 Research Unit for Molecular Medicine, Skejby Sygehus, DK 8200, Århus N, Denmark; 4 Institute of Human Genetics, Aarhus University, Aarhus, Denmark; 5 Faculty of Medicine, University of Oslo, Norway

## Abstract

**Background:**

2-methylbutyryl-CoA dehydrogenase deficiency or short/branched chain acyl-CoA dehydrogenase deficiency (SBCADD) is caused by a defect in the degradation pathway of the amino acid L-isoleucine.

**Methods:**

We report a four-year-old mentally retarded Somali boy with autism and a history of seizures, who was found to excrete increased amounts of 2-methylbutyryl glycine in the urine. The SBCAD gene was examined with sequence analysis. His development was assessed with psychometric testing before and after a trial with low protein diet.

**Results:**

We found homozygosity for A > G changing the +3 position of intron 3 (c.303+3A > G) in the SBCAD gene. Psychometric testing showed moderate mental retardation and behavioral scores within the autistic spectrum. No beneficial effect was detected after 5 months with a low protein diet.

**Conclusion:**

This mutation was also found in two previously reported cases with SBCADD, both originating from Somalia and Eritrea, indicating that it is relatively prevalent in this population. Autism has not previously been described with mutations in this gene, thus expanding the clinical spectrum of SBCADD.

## Background

2-methylbutyryl-CoA dehydrogenase deficiency, also known as short/branched chain acyl- CoA dehydrogenase deficiency (SBCADD) (OMIM 61006), is a recently described autosomal recessive disorder caused by a defect in the degradation pathway of L- isoleucine leading to increased urinary excretion of 2-methylbutyryl glycine [1,2]. The enzymatic defect results from disruption of the SBCAD gene (*ACADSB*) [3]. The clinical consequences of mutations in this gene are unclear as patients can be divided into two categories. The first category consists of infants detected by newborn screening programmes. These infants are treated with diet and remain without clinical symptoms. In the second category affected patients were diagnosed because they presented clinically with seizures and psychomotor delay and had increased urinary excretion of 2-methylbutyryl glycine [4,5]. The brain, where isoleucine is oxidized as fuel, is primarily affected in these patients. We report an autistic and mentally retarded boy with 2-methyl butyryl glycinuria and homozygosity for a deleterious mutation in *ACADSB*. He is the first to be described with SBCADD and autism.

## Case presentation

The patient was a four-year-old boy who was the third child of healthy Somali parents. They belong to the same tribe, but are probably not closely related. After an uneventful pregnancy, he was delivered at term without complications in Somalia in 2001. Birth weight, length, head circumference and Apgar scores are not known, but the mother described him as a healthy and normal infant until he was 5 months old. From this age he started to have seizure-like movements, which according to the mother's description, could have been myoclonic epilepsy or infantile spasms. At first, the seizures appeared at night, but soon they also occurred during the day, with increasing frequency and duration. The seizures stopped at 8 months after he was examined in a local hospital and put on antiepileptic drug therapy. Despite becoming seizure free, the mother described him as becoming markedly less responsive and alert.

Growth and motor development were normal for his age. He crawled at the age of 9 months and walked unsupported at the age of 12 months. Speech development was significantly delayed. The family moved to Norway when he was 1 1/2 years old. Although he could say some words at 2 years of age, verbal language skills improved only little after this age. At the age of 4 years he entered a day-care centre for autistic children, as it was observed that he had limited communication and social skills, gave poor eye contact and displayed a repetitive pattern of behavior.

Clinical examination at 5 years did not reveal any dysmorphic features. His muscle tone and strength and coordination and deep tendon reflexes were normal without signs of peripheral sensory deficit. His height and weight corresponded to the 25^th ^centiles, and his head circumference was at the 75^th ^centile. Eye examination with fundoscopy, hearing test, electroencephalography (EEG), echocardiography of the heart and magnetic resonance imaging of the brain were normal. Blood glucose, urea, creatinine, and liver transaminases were normal. Chromosome analysis showed a normal 46, XY karyotype. A Southern blot test for Angelman syndrome was negative.

Since the time of the diagnosis of SBCADD our patient was on a trial with protein restricted diet (1 g protein/kg/day) for 5 months. The diet was reasonably well adhered to. He was tested by a psychologist using standard tests before and after 5 months of protein restriction treatment. No observable improvement was demonstrated during the period.

### Neuropsychological examination

When examined with ADOS-G (Autism Diagnostic Observation Schedule-Generic), he obtained a score of 13, just above the cut-off score of 12 for childhood autism [6]. ADI-R (Autism Diagnostic Interview-Revised) could not be administered, due to his mother's limited knowledge of the Norwegian language. When tested with Bayley Scale of Infant Development at 4 years, his cognitive ability score corresponded to that of 2 years. Like most autistic children, his visual-spatial abilities were much better than his language abilities. WPPSI-R (Wechsler Preschool and Primary Scale of Intelligence) at 5 years demonstrated visual-spatial abilities corresponding to 3 years of age. The verbal tests could not be administered due to lack of verbal abilities. Psychometric testing concluded with moderate mental retardation. This was also in accordance with the Vineland-2 Adaptive Behavior Scale, reported by his teacher. Motor development appeared normal when using the Movement Assessment Battery for Children test manual. During this examination, he was not able to follow verbal instructions.

### Biochemical examinations

Because mental retardation and autism sometimes can be caused by inborn errors of metabolism [7], plasma and urine samples from the patient were analyzed by a metabolite screening system [8] including gas chromatography-mass spectrometry (GC/MS) (organic acids), amino acid analyzer, tandem mass spectrometry (MS/MS) (purines, pyrimidines, acylcarnitines), and capillary electrophoresis (transferrin variants). Considerable amounts of 2-methylbutyryl glycine were identified in his urine. The excretion of 2-ethylhydracrylic acid was unremarkable. The patterns of amino acids and purines/pyrimidines were normal, except for a slight increase in glycine. MS/MS analysis of acylcarnitines showed increased plasma level of C5-acylcarnitine (1.43 micromoles/L, reference range 0.05–0.38) and an increased C5/C3-acylcarnitines ratio of 2.83 (reference range 0.16–0.73).

### Genetic analysis

DNA was extracted from blood and cultured fibroblasts by standard methods. PCR amplification of all exons and part of the flanking intron sequences of the human ACADSB (SBCAD) gene were carried out by use of intron-located primers under standard conditions in an automated thermal cycler (Thermal cycler 480, PE) [1]. The PCR products were sequenced in both directions using a 3100-Avant genetic analyzer and a BigDye^® ^Terminator v1.1 Cycle Sequencing kit (Applied Biosystems). Sequence analysis of all exons, and part of the flanking introns of the human SBCAD gene from patient genomic DNA, revealed an apparent homozygosity for an A > G mutation, which changed the +3 position of intron 3 of the SBCAD gene (c.303+3A > G). No other changes were observed. Sequence analysis of the mother showed heterozygosity for the same c.303+3A > G mutation.

## Discussion

It is well known that inborn errors of metabolism can cause developmental delay, epilepsy and various other neurological symptoms. The yield of metabolic examinations increase with the severity of developmental delay and the extent of associated neurological handicaps. In routine clinical practice, metabolic, genetic, and neuroimaging investigations are indicated in selected children. Abnormalities uncovered by such studies rarely dictate significant change in the management of those patients, but may provide important information for family members. Novel evidence on brain mechanisms may occasionally advance our understanding of the brain dysfunctions responsible for the phenotype. The association of SBCADD with autism may be causative or possibly a chance association given the frequency of autism in the general population.

Because many of these biochemical defects can be indicated by analysis of organic acids in urine and MS/MS-based analysis of acyl-carnitines in blood-spots or plasma, we have initiated a program with metabolic screening from autistic patients at our hospital. So far we have screened approximately 80 individuals with autistic spectrum disorder since 2004. The patient in the present study had increased urinary excretion of 2-methylbutyryl glycine. He also had increased C5-carnitine levels in plasma, which can be caused by isovaleric acidemia, antibiotics (Pivalic) or SBCADD. Based on these biochemical findings, SBCADD was suspected as the most likely condition and the diagnosis was confirmed by identification of homozygosity for the c.303+3A > G mutation. The c.303+3A > G mutation causes complete skipping of exon 3 and the deleterious nature of this mutation has previously been confirmed in patient cells and also from minigene studies [5]. The same mutation was identified in two other unrelated East-African (Ethiopia and Somalia) families affected by SBCADD. One patient was homozygous and another patient was predicted to be compound heterozygous for the mutation [2]. The fact that the present patient is also of East-African origin may indicate that the c.303+3A > G mutation is relatively prevalent in this geographical area.

Several amino acids are involved in the process of neuronal signalling, either as neurotransmitters, or they act as precursors or antagonists of neurotransmitters. Disturbance in the metabolic pathway of these amino acids as occurring in SBCADD, could therefore be predicted to cause a wide range of neurological symptoms, for example seizures, mental retardation and autism. The three amino acids with branched side chains, leucine, isoleucine and valine are oxidized as fuels in the brain, although their primary role is in muscle, adipose tissue, and kidney.

Our patient exhibited clinical signs of brain dysfunction including mental retardation, abnormal behavior, and a history of infantile seizures. We have no record of the EEG findings from the time he was hospitalized in Somalia, so the question regarding possible hypsarrythmia and infantile spasms is unanswered. However, this epileptic syndrome was most likely the case, considering a typical age of onset and description of the seizures. Infantile spasms also occurred in the SBCADD patient reported by Madsen [5] and epilepsy was reported in the patient described by Gibson [2], although the seizures were described as being focal.

Our patient exhibited developmental abnormalities compatible with autism, thus expanding the clinical spectrum of this rare biochemical disorder. Developmental regression has so far not been observed in SBCADD. However, a defect in a neighbouring enzyme step in the degradation pathway of isoleucine, 3-hydroxy-2-methylbutyryl-CoA dehydrogenase deficiency (OMIM 300438), was characterised by progressive dystonia, seizures and blindness [9,10].

In inherited metabolic disorders, homozygosity for a particular disease causing mutation is not always sufficient to cause clinical symptoms. The mother of one clinical case and the sister of another case were shown to have genetic defects identical to their SBCADD relatives. In addition, they excreted 2-methylbutyrylglycine in the urine. Despite the genetic and biochemical abnormalities, these relatives were asymptomatic [2,5].

Because SBCADD may cause accumulation of C5-carnitine in the blood, the enzyme defect can be detected by MS/MS analysis of blood spots in the screening of newborns for metabolic disease [11]. Using this method, 8 Hmong Chinese neonates were identified to have moderately elevated plasma C5-acylcarnitine levels and were subsequently found to have 2-methylbutyryl glycinuria. Molecular genetic analysis in 3 of the cases revealed homozygosity for a c.1165A > G mutation, which is associated with skipping of exon 10 of the SBCAD gene. Except for one child who developed mild muscle hypotonia, the other cases were asymptomatic. The explanation for a mild phenotype in these individuals of Hmong Chinese origin is unclear. Possibly, overlapping enzyme activity from other short branched acyl-CoA enzymes, such as ACAD-8, with SBCAD may be sufficient to avoid harmful accumulation of metabolites from isoleucine metabolism in patients with SBCADD under circumstances when they are not metabolically challenged [1]. External factors such as infections and metabolic stress may be necessary to trigger disease symptoms in genetically predisposed individuals. For example, in other acyl-CoA dehydrogenase defects, such as MCAD and glutaryl-CoA dehydrogenase deficiency, asymptomatic homozygous relatives of clinically affected individuals have been reported [12-14].

Deficiency of SBCAD leads to accumulation of its substrate, 2-methylbutyryl-CoA within the mitochondrion. This substance is transesterified with glycine by the mitochondrial enzyme acyl-CoA glycine-*N*-acyltransferase (glycine-*N*-acylase) to form 2-methylbutyryl glycine. The discovery of SBCADD in the first reported patients, as well as in our patient, relies on the detection of abnormal excretion of 2-methylbutyryl glycine in the urine. The frequency of SBCADD is probably underestimated because detection and recognition of urine acylglycines is problematic. 2-ethylhydracrylic aciduria has been proposed as a new diagnostic marker that could lead to improved detection of SBCAD [15], but the excretion of this acid was not remarkable in our patient. Also, a high normal acylcarnitine profile with C5 carnitine below the cut off value as previously described in two patients, suggests that SBCADD may go undetected and hence be more frequent than indicated from the limited number of reported cases [11].

## Conclusion

It is important to consider an underlying metabolic defect in children presenting with autism, particularly when accompanied by seizures and severe developmental delay. The diagnosis of SBCADD in an autistic Somali patient was established by the detection of 2-methylbutyryl glycine, using GS/MS screening of the urine, and was confirmed by demonstrating homozygosity for c.303+3A > G change, which may be more frequent in Somalia and Eritrea. We suggest that autism spectrum disorder should be included in the phenotypic spectrum of SBCADD.

## Abbreviations

ACADSB: acyl-CoA dehydrogenase short/branched chain

GC/MS: gas chromatography – mass spectrometry

MS/MS: tandem mass spectroscopy

SBCADD: short branched chain acyl CoA dehydrogenase deficiency

## Competing interests

The author(s) declare that they have no competing interests.

## Authors' contributions

All authors have read and approved the manuscript. OJK diagnosed the patient and drafted the initial manuscript, BW and JE performed the biochemical analyses, BT evaluated the patient with psychometric testing, BSA performed the molecular analysis and contributed to drafting the manuscript, and PS initiated the study and drafted the manuscripts.

## References

[B1] Andresen BS Christensen E Corydon TJ Bross P Pilgaard B Wanders RJ Ruiter JP Simonsen H Winter V Knudsen I Schroeder LD Gregersen N Skovby F Isolated 2-methylbutyrylglycinuria caused by short/branched-chain acyl-CoA dehydrogenase deficiency: identification of a new enzyme defect, resolution of its molecular basis, and evidence for distinct acyl-CoA dehydrogenases in isoleucine and valine metabolism Am J Hum Genet 2000 67 1095 1103 10.1086/303105 11013134 PMC1288551

[B2] Gibson KM Burlingame TG Hogema B Jakobs C Schutgens RB Millington D Roe CR Roe DS Sweetman L Steiner RD Linck L Pohowalla P Sacks M Kiss D Rinaldo P Vockley J 2-Methylbutyryl-coenzyme A dehydrogenase deficiency: a new inborn error of L-isoleucine metabolism Pediatr Res 2000 47 830 833 10.1203/00006450-200006000-00025 10832746

[B3] Rozen R Vockley J Zhou L Milos R Willard J Fu K Vicanek C Low-Nang L Torban E Fournier B Isolation and expression of a cDNA encoding the precursor for a novel member (ACADSB) of the acyl-CoA dehydrogenase gene family Genomics 1994 24 280 287 10.1006/geno.1994.1617 7698750

[B4] Korman SH Inborn errors of isoleucine degradation: a review Mol Genet Metab 2006 89 289 299 10.1016/j.ymgme.2006.07.010 16950638

[B5] Madsen PP Kibaek M Roca X Sachidanandam R Krainer AR Christensen E Steiner RD Gibson KM Corydon TJ Knudsen I Wanders RJ Ruiter JP Gregersen N Andresen BS Short/branched-chain acyl-CoA dehydrogenase deficiency due to an IVS3+3A>G mutation that causes exon skipping Hum Genet 2006 118 680 690 10.1007/s00439-005-0070-4 16317551

[B6] Lord C Risi S Lambrecht L Cook EH Jr. Leventhal BL DiLavore PC Pickles A Rutter M The autism diagnostic observation schedule-generic: a standard measure of social and communication deficits associated with the spectrum of autism J Autism Dev Disord 2000 30 205 223 10.1023/A:1005592401947 11055457

[B7] Stromme P Diseth TH Prevalence of psychiatric diagnoses in children with mental retardation: data from a population-based study Dev Med Child Neurol 2000 42 266 270 10.1017/S0012162200000451 10795566

[B8] Jellum E Kvittingen EA Thoresen O Guldal G Horn L Seip R Stokke O Systematic laboratory diagnosis of human metabolic disorders Scand J Clin Lab Invest Suppl 1986 184 11 20 3473607

[B9] Ensenauer R Niederhoff H Ruiter JP Wanders RJ Schwab KO Brandis M Lehnert W Clinical variability in 3-hydroxy-2-methylbutyryl-CoA dehydrogenase deficiency Ann Neurol 2002 51 656 659 10.1002/ana.10169 12112118

[B10] Sutton VR O'Brien WE Clark GD Kim J Wanders RJ 3-Hydroxy-2-methylbutyryl-CoA dehydrogenase deficiency J Inherit Metab Dis 2003 26 69 71 10.1023/A:1024083715568 12872843

[B11] Matern D He M Berry SA Rinaldo P Whitley CB Madsen PP van Calcar SC Lussky RC Andresen BS Wolff JA Vockley J Prospective diagnosis of 2-methylbutyryl-CoA dehydrogenase deficiency in the Hmong population by newborn screening using tandem mass spectrometry Pediatrics 2003 112 74 78 10.1542/peds.112.1.74 12837870

[B12] Andresen BS Bross P Udvari S Kirk J Gray G Kmoch S Chamoles N Knudsen I Winter V Wilcken B Yokota I Hart K Packman S Harpey JP Saudubray JM Hale DE Bolund L Kolvraa S Gregersen N The molecular basis of medium-chain acyl-CoA dehydrogenase (MCAD) deficiency in compound heterozygous patients: is there correlation between genotype and phenotype? Hum Mol Genet 1997 6 695 707 10.1093/hmg/6.5.695 9158144

[B13] Korman SH Gutman A Brooks R Sinnathamby T Gregersen N Andresen BS Homozygosity for a severe novel medium-chain acyl-CoA dehydrogenase (MCAD) mutation IVS3-1G > C that leads to introduction of a premature termination codon by complete missplicing of the MCAD mRNA and is associated with phenotypic diversity ranging from sudden neonatal death to asymptomatic status Mol Genet Metab 2004 82 121 129 10.1016/j.ymgme.2004.03.002 15171999

[B14] Waddell L Wiley V Carpenter K Bennetts B Angel L Andresen BS Wilcken B Medium-chain acyl-CoA dehydrogenase deficiency: genotype-biochemical phenotype correlations Mol Genet Metab 2006 87 32 39 10.1016/j.ymgme.2005.09.020 16291504

[B15] Korman SH Andresen BS Zeharia A Gutman A Boneh A Pitt JJ 2-ethylhydracrylic aciduria in short/branched-chain acyl-CoA dehydrogenase deficiency: application to diagnosis and implications for the R-pathway of isoleucine oxidation Clin Chem 2005 51 610 617 10.1373/clinchem.2004.043265 15615815

